# Bonding widths of Deposited Polymer Strands in Additive Manufacturing

**DOI:** 10.3390/ma14040871

**Published:** 2021-02-11

**Authors:** Cheng Luo, Manjarik Mrinal, Xiang Wang, Ye Hong

**Affiliations:** Department of Mechanical and Aerospace Engineering, University of Texas at Arlington, Arlington, TX 76010, USA; manjarik.mrinal@mavs.uta.edu (M.M.); xiang.wang@mavs.uta.edu (X.W.); ye.hong@mavs.uta.edu (Y.H.)

**Keywords:** fused-filament additive manufacturing, deposition deformation, polymer melt, high viscosity, cross-sectional profile, bonding width, modelling

## Abstract

In this study, we explore the deformation of a polymer extrudate upon the deposition on a build platform, to determine the bonding widths between stacked strands in fused-filament fabrication. The considered polymer melt has an extremely high viscosity, which dominates in its deformation. Mainly considering the viscous effect, we derive analytical expressions of the flat width, compressed depth, bonding width and cross-sectional profile of the filament in four special cases, which have different combinations of extrusion speed, print speed and nozzle height. We further validate the derived relations, using our experimental results on acrylonitrile butadiene styrene (ABS), as well as existing experimental and numerical results on ABS and polylactic acid (PLA). Compared with existing theoretical and numerical results, our derived analytic relations are simple, which need less calculations. They can be used to quickly predict the geometries of the deposited strands, including the bonding widths.

## 1. Introduction

**Figure 1:**
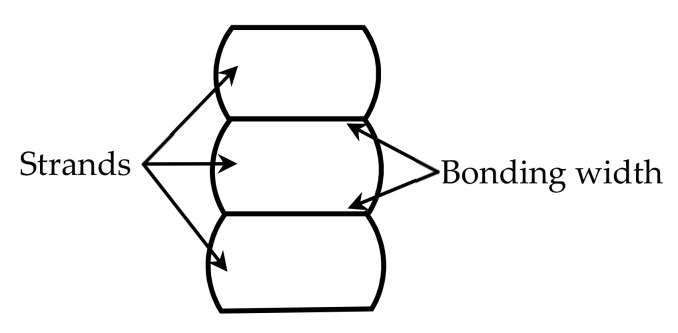
Stack of deposited strands for the formation of a 3D plastic structure (cross-sectional illustration).

Fused-filament fabrication (FFF), also known as fused deposition modeling or material extrusion additive manufacturing, is one of the most widely used processes in additive manufacturing for fabricating three-dimensional (3D) plastic structures [1]. In FFF, deposited strands are stacked together to form a plastic structure (Figure 1). The bonding strength between the stacked strands is one of the important factors that determine the strength of the fabricated plastic product. In addition to the temperatures of the filaments [2], the bonding width between the cross-sections of two neighboring strands also affects the bonding strength (Figure 1). A large bonding width leads to a large bonding area, and thus a high bonding strength. As such, it is important to know the relation between the bonding width and processing parameters (such as extrusion and print speed).

**Figure 2:**
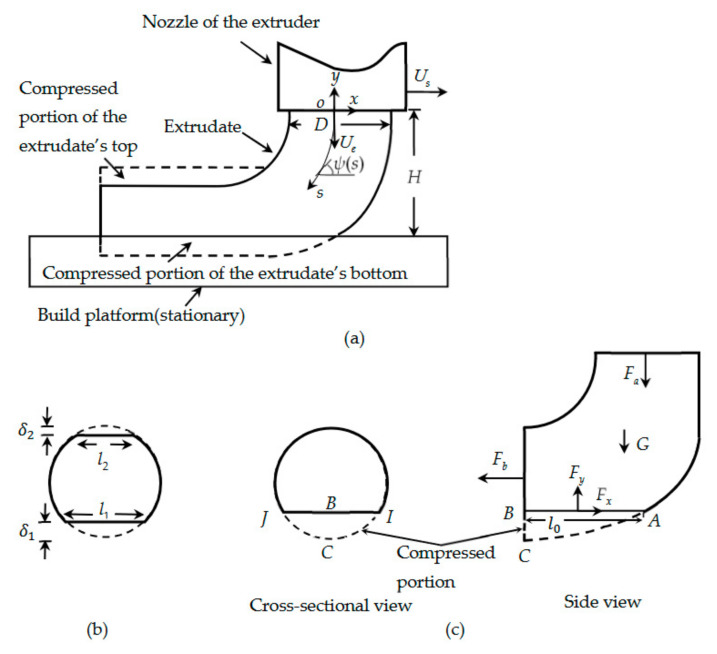
Deposition of an extrudate on a stationary build platform: (**a**) side view of the setup, (**b**) cross-sectional view of the compressed extrudate during the collision, and (**c**) free-body diagram of the extrudate (nonscaled).

In FFF, a solid filament is melted in an extruder, extruded from its nozzle, and then deposition is carried out on a build platform (Figure 2a). Several analytical models have been developed to understand the melting processes and melt flows inside extruders. A case was considered in [3], where the filament melted as soon as it entered the extruder. A limiting case was investigated in [4], where a solid filament only began to melt when it reached near the end of the extruder [4]. The effects of feed rate on the temperature profile and feed force were considered in [5]. The upper bound of feed rates was determined in [6] that did not cause jamming during the extrusion.

During the deposition, the cross-section of an extrudate is deformed when the extrudate contacts the build platform (Figure 2a). This deformation decides the bonding width. Previously, extensive studies have been carried out on the deformation of an object in its contact region with another object, such as a solid sphere or cylinder pressed against a solid substrate [7], water drop resting on a flat surface [8], water drop rolling on an inclined surface [8], drops of n-heptane [9] and water [10] impacting solid substrates, and molten particles of tin [11], alumina [12], and lead [13] colliding with cold surfaces.

Recently, other researchers have done much work to specifically explore the deposition process in FFF, through numerical simulation [14,15,16,17] and theoretical analysis [18]. They have explored the effects of the speed ratio [14,15], viscosity [15], viscoelasticity [16], deposition flows [17], and interlayer contact pressures [18] on the dimensions of the deposited strands for particular combinations of processing parameters. As indicated in [19], a simple method is needed to calculate the geometries of the deposited filament for FFF. For this purpose, an empirical formula was proposed in [19] to predict the widths of the deposited strands. Naturally, a rigorously derived formula, which involves less empirical hypotheses, should give a better prediction.

The liquids considered in [8,9,10,11,12,13] have dynamic viscosities smaller than 1 Pa s [20]. Therefore, the viscous effect is not dominant over effects such as gravity [8], inertia [9], and surface tension [10]. In contrast, the filament materials commonly used in FFF, such as acrylonitrile butadiene styrene (ABS), polylactic acid (PLA), and polylactic acid-polyhydroxybutyrate copolymer (PLA-PHB), have much higher viscosities at the temperatures that they are extruded [14,15,21,22]. The viscosities further increase with the decrease in temperature during the cooling of the extrudates. Consequently, the deformations of these extrudates should be more affected by viscous forces. The analysis of the corresponding deformation may be simplified by comparing the viscous effect with the other effects, such as those of surface tension, inertia, and gravity. This comparison may enable the identification of both significantly large and negligibly small factors on the deformation. Accordingly, it is used in the present study to help analyze the deformation of the deposited strands in FFF.

Based on this analysis, our aim is to derive simple theoretical relations, which can be used to quickly predict the geometries of the deposited filaments for different combinations of processing parameters, such as extrusion speed, print speed and nozzle height. Particularly, they can be applied to determine the combination of the processing parameters that may lead to a large bonding width.

## 2. Materials and Experimental Methods

Experiments are performed using ABS to validate the derived relations. Single strands are printed with white ABS filaments (MakerBot Company, Brooklyn, USA), using a Creality Ender Pro 3D printer (Creality 3D Technology Company, Shenzhen, China). The nozzle employed in the printer has an opening with a diameter of 0.4 mm. Temperature of printing platform and nozzle are maintained at 90 °C and 235 °C throughout the prints. The platform is kept at a temperature lower than glass-transition temperature for ABS as of 104.5 °C [2], to avoid the effect of the bed temperature on the strands’ cross-sections.

A cuboidal wire model with a length of 120 mm is designed in software SOLIDWORKS and exported as STL file. Simplified 3D is used as the slicing software to import the STL file of the computer aid design model, and also to generate G-code embodying all printing parameters. The print speed ($U_{s}$), which refers to the horizonal speed of the nozzle head, is set as 10 mm/s in the software. The extrusion speed ($U_{e}$), which is the speed that the polymer melt exits the nozzle, is calculated according to the law of mass conservation: $U_{e}$ = *Q*/*A_d_*, where *Q* denotes the amount of extruded material per unit time and *A_d_* is the measured cross-sectional area of the deposited filament.

The deformations of extrudates are considered in four special cases, which will be discussed in Section 4. In each case, the layer height input in the printer setting defines the nozzle height. As designed, a printed strand is 120 mm long. Its length is kept long enough to ensure that it has a uniform extrusion, primarily focusing on the mid-section. The strand is cut at the middle using a brand-new razor blade with one stroke. The cutting is square to the printing direction to expose its cross-section for analysis. No deformation is observed along the cutting edge that is big enough to alter the experimental result. Three repetitions are performed for each test and their results are used to calculate standard deviation. Average value of all measurements is taken as experimental value.

**Figure 3:**
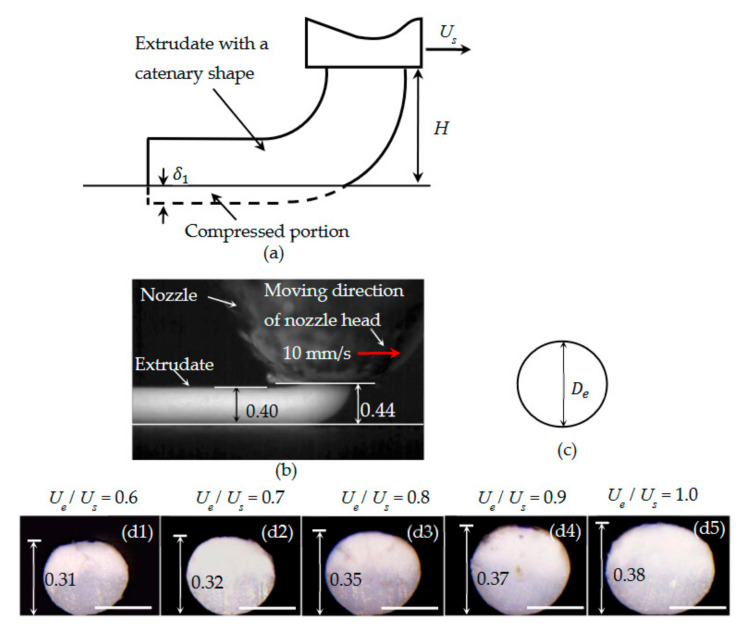
(**a**) Illustration of Case I.1. (**b**) A representative example, in which an acrylonitrile butadiene styrene (ABS) filament was extruded through a nozzle with diameter 0.40 mm, when *H* = 0.44 mm and $U_{e}$ = $U_{s}$ = 10 mm/s. (**c**) Predicted circular cross-section of a deposited strand. (**d1**–**d5**) Cross-sections of the deposited strands, with *H* fixed to be 0.5 mm and *U_e_ /U_s_* ranging from 0.6 to 1.0. Scale bar: 0.25 mm. The units in (**d1**–**d5**) are all mm, which also applies to all of the following figures.

**Figure 4:**
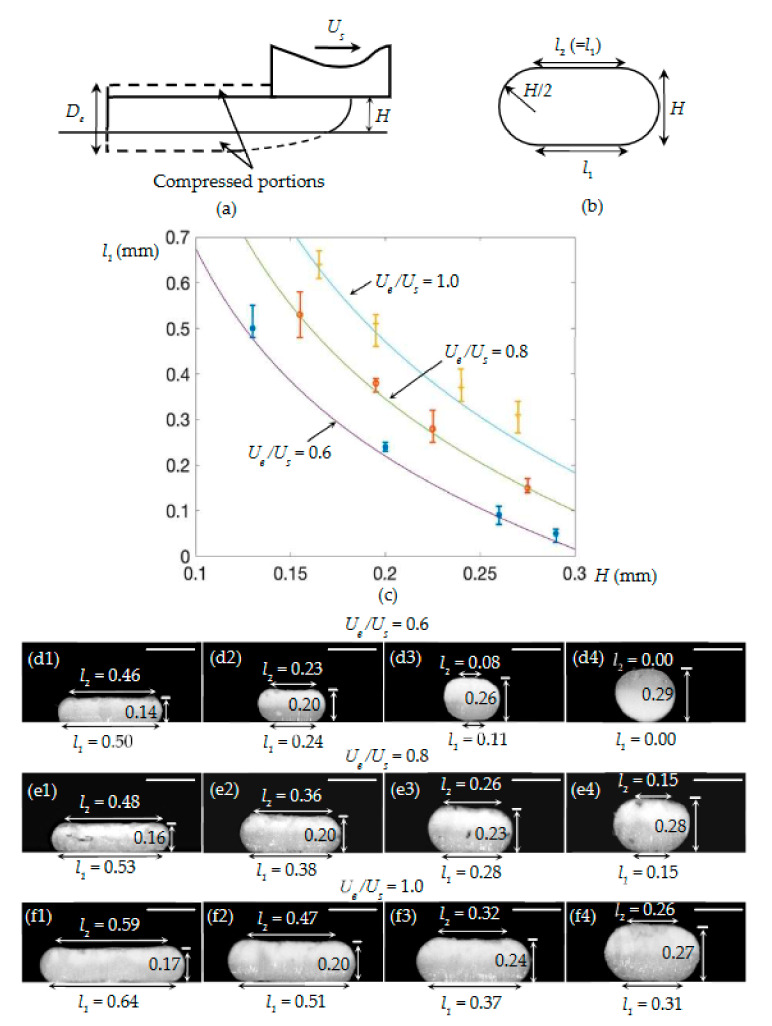
(**a**) Illustration of Case I.2. (**b**) Predicted cross-section of a deposited strand. (**c**) Theoretical and experimental *H*-*l_1_* relations. (**d1**–**f4**) Representative cross-sections of the deposited strands, when *U_e_*/*U_s_* ranged from 0.6 to 1.0 for various nozzle heights. Scale bar: 0.30 mm.

**Figure 5:**
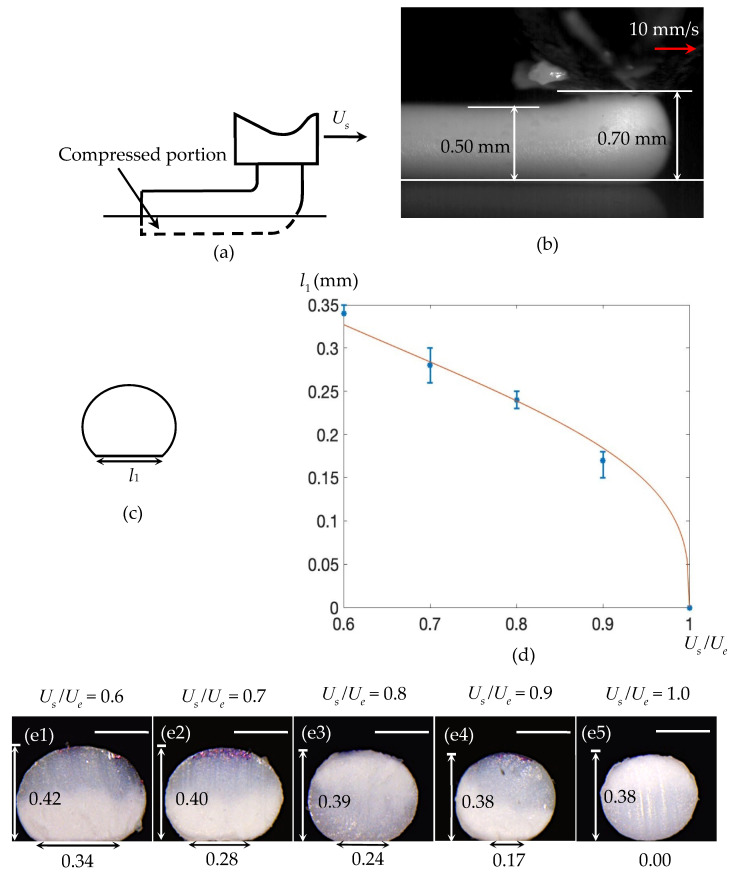
(**a**) Illustration of Case II.1. (**b**) A representative example, in which an ABS strand was extruded through a nozzle with diameter 0.40 mm when *H* = 0.70 mm, $U_{e}$ = 16.5 mm/s and $U_{s}$ = 10 mm/s. (**c**) Predicted cross-section of a deposited strand. (**d**) Theoretical and experimental results. The curve denotes the theoretical prediction obtained using Equation (48). (**e1**–**e5**) Cross-sections of the deposited strands, when *H* was fixed to be 0.50 mm and *U_s_ /U_e_* ranged from 0.6 to 1.0. Scale bar: 0.25 mm.

**Figure 6:**
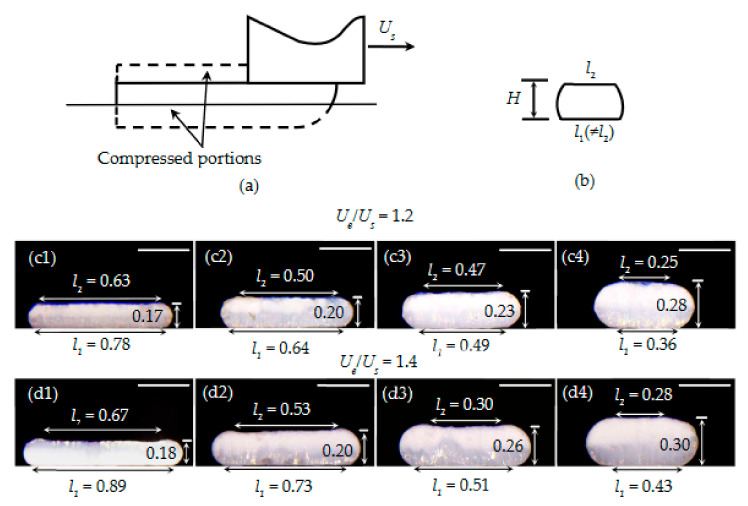
(**a**) Illustration of Case II.2. (**b**) Cross-sectional view of a compressed strand. (**c1**–**d4**) Representative cross-sections of the deposited strands, when the *U_e_ /U_s_* ratios are 1.2 and 1.4 at different nozzle heights. Scale bar: 0.25 mm.

A Dino-Lite Pro Digital Microscope (AnMo Electronics Corporation, Taipei, Taiwan) is utilized to take a cross-sectional picture along the axial direction of the strand (Figure 3d, Figure 4d, Figure 5e and Figure 6c1–d4).

The contour of a strand’s cross-section is used to evaluate its area from the image captured via software AutoCAD. The height of each strand is measured manually with a digital caliper, which has a measurement error of 1 *µ*m. This height is also used to calibrate the image. At the time of measuring a strand, it has already cooled down to a room temperature. As such, it is rigid, and has negligible elastic deformation during the measurement. Accordingly, the measurement error induced by the elastic deformation is negligible, in comparison with the cross-sectional dimensions of a strand, such as height and width, which are all over 100 *µ*m.

A high-speed camera, Fastec TS3 (Fastec Imaging Company, San Diego, USA), is installed to record the deposition processes at 500 fps. Figure 3b and Figure 5b are extracted from the recorded videos. The corresponding images are calibrated according to the nozzle height.

## 3. Model

To release the residual stress induced during an extrusion, an extrudate may start to swell at the nozzle exit. This expansion is referred to as the die-swell effect. The swell ratio and terminal swell distance are parameters used to characterize this effect. The former parameter is the ratio between the diameters of the nozzle opening and swelled extrudate, while the latter is the distance where the maximum swell diameter is attained. These two parameters depend on the type of the used polymer, nozzle length/diameter ratio, and shear rate in the nozzle [23,24,25,26].

By default, the solid filaments currently used in FFF, such as those used in commercial 3D printers, have the same diameter of 1.75 mm. The nozzles normally have circular openings, with diameters in the range of 0.2 to 0.8 mm. Accordingly, the extrudate is considered to have a circular cross-section. The length/diameter ratio of the nozzle varies from one to five. The feed rate of a solid filament is in the order of 1 mm s^−1^ [1]. According to the law of conservation of mass, an extrudate near the nozzle exit has a speed in the order of 10 mm s^−1^, as shown in the printing of ABS filaments [2]. The corresponding shear rate in the nozzle is in the order of 10 s^−1^.

Using a typical FFF extruder with this shear rate, we measured the swell ratios and terminal swell distances of extruded ABS filaments. The swell ratios varied between 1.05 and 1.10, while the terminal swell distances were smaller than 20% of the nozzle diameter. According to these data, even when the die-swell effect is considered, the extruded strand in FFF may have a diameter of the same order of magnitude as that of the nozzle opening before its deposition on the substrate. We denote this diameter as *D*. Owing to the relaxation of the restraint of the nozzle and negligible air drag on its surface, the speed of the extrudate becomes uniform across its cross-section after it travels the terminal swell distance [24,27]. As *D* is in the same order of magnitude as that of the nozzle opening, this speed is in the same order of magnitude as $U_{e}$.

Consider that the extrudate falls in a steady shape onto a stationary platform. We set up a rectangular *x − y* coordinate system, as defined in Figure 2a. *H* denotes the distance between nozzle head and build platform, *s* is the arc length measured from the exit of the nozzle, *ψ*(*s*) is the inclination of the centerline to the horizontal, and $U\left(s\right)$ is the speed of the extrudate (Figure 2a). At the exit of the nozzle, *ψ* (0) $=\frac{\pi }{2}$ and $U\;\left(0\right)$ = $U_{e}$.

The polymer melt is modelled as a power-law non-Newtonian fluid [3,21,28,29,30,31]. During the deposition process, an extrudate may be compressed. We assume that the elongation viscosity, *µ*, has a power-law relation under the generalized Newtonian fluid (GNF) framework. Equation (1).

(1) $$µ=L{\left|\frac{dU}{ds}\right|}^{m-1}$$

where *L* and *m* are the extensional consistency and thinning (or thickening) indices, respectively. *L* changes with temperature. When *m* = 1, Equation (1) is reduced to the constitutive relation for a Newtonian fluid. Let *K* and *n* denote the shear consistency and thinning (or thickening) indices, respectively. As in [32], for simplicity, we also assume that *m = n*. Define the Trouton ratio, *Tr* = *L/K*. Within the GNF framework, *Tr* = 3, indicating that *L* is in the same order as *K*. For PLA, *n* = 0.433, and *K* = 3.54 × 10^4^ Pa s*^n^* at 170 °C (Table I of [32]). In the case of ABS, *n* = 0.32, and *K* = 1.04 × 10^4^ Pa s*^n^* at 230 °C [2]. 170 °C and 230 °C are extrusion temperatures that have been used to print PLA [32] and ABS [2], respectively.

According to the above discussion and material properties of the filaments used in FFF, Equation (2) [1,2,4,5,6,7,8,9,10,11,12,13,14,15,16,17,18,19,20,21,22,23,24,25,26,27,28,29,30,31,32,33].

(2) $$D\;\sim \;0.5\;\mathrm{mm},\;U_{e}\sim \;10\;\mathrm{mm}\;s^{-1},\;L\;\sim \;10000{\;\mathrm{Pa}\;s}^{m},\;\gamma \;\sim \;10\;\mathrm{mN}\;m^{-1},\;\rho \;\sim \;1000\;\mathrm{kg}\;m^{-3}$$

where γ and ρ are surface tension and mass density of the polymer melt, respectively, and *L* is estimated at the extrusion temperature.

The bonding is considered to occur mainly above the melting or glass-transition temperature. Within 2 s, the ABS melt was cooled from the extrusion temperature of 260 °C to its glass-transition temperature of 104.5 °C [2,34]. To ensure a larger bonding time, the travel time of the extrudate from the nozzle exit to the substrate has to be considerably smaller than 2 s. In addition, two results of [2] are important. First, the cooling rate of the ABS extrudate is 10 °C per 0.1 s for the first second. Second, the viscosity of the ABS melt rapidly increases with the decrease in temperature. Therefore, to reduce the increase in the viscosity as well as to increase the bonding time, it is reasonable to limit the above travel time to values smaller than 0.1 s. Considering Equation (2), this time limit implies that the nozzle height should be smaller than 1 mm. In other words, H scales as 0.5 mm and $\frac{H}{D}$ is less than 2.

We consider that, when a strand just leaves the nozzle, it has a circular cross-section. Let $\delta_{1}$ and $l_{1}$, respectively, denote the compressed depth and flat width of the deposited strand’s bottom surface (Figure 2b). $\delta_{2}$ and $l_{2}$ are the counterparts of $\delta_{1}$ and $l_{1}$ at the top surface of the deposited strand. $l_{b}$ is the smaller value between $l_{1}$ and $l_{2}$, and it is the bonding width between two stacked filaments.

An extruded strand may impact a solid surface in a manner similar to that a viscous thread [27,33,34,35,36,37,38] or sheet [38] falls from a nozzle onto a horizontal surface. The previous studies focused on the shapes and dynamics of the falling threads or sheets, but not on their deposition deformations. In addition, golden syrup was the liquid having the highest viscosity in the previous studies [27,33,34,35,36,37,38]. Its viscosity is in the order of 10 Pa s [27], which is at least three orders of magnitude lower than those of its counterparts in FFF. This difference also indicates that the viscous effect should be more dominant in FFF.

Considering these results and previous modelling studies on the viscous threads and sheets [27,33,34,35,36,37,38], particularly that of [27], in the present study we investigate the deformations of the extruded strands at their interfaces with the substrates. As will be detailed in the rest of this section, as well as in Section 4, we begin this investigation with the theoretical model of [27], which was originally developed for a Newtonian fluid. We extend it to consider a non-Newtonian fluid, and then simplify it by mainly considering the viscous effect. Finally, with the aid of the derived formulas and according to the balance of linear momentum and also the balance of energy, we explore the deposition deformation, obtaining theoretical relations for predicting the geometries of the deposited strands.

The following axial and transverse equations of motion were derived in [27] for a viscous strand based on the extensional-flow approximation, Equations (3) and (4):

(3) $$\rho \pi D^{2}U\frac{dU}{ds}=4\frac{dF}{ds}-\pi D^{2}\rho g\sin\psi$$

(4) $$\rho \pi D^{2}U^{2}\frac{d\psi }{ds}=4F\frac{d\psi }{ds}-\pi D^{2}\rho g\cos\psi$$

where *F* is the axial force acting over the cross-section of the suspended strand. The viscous resistance to stretching or compression was considered in these two equations, while the viscous resistance to shear deformations was neglected. The shear deformations might be caused by the bending and air drag, which were neglected in [27]. It was also indicated in [27] that the neglect of the strand’s bending precluded the buckling mechanism. Since the nozzle height considered in the present study is not large, the buckling does not occur. As such, the bending deformation is not large. Meanwhile, the drag force of the surrounding air is negligible, due to the small density of air and relatively low extrusion speed. Therefore, Equations (3) and (4) still apply to the present study to consider the shape and dynamics of an extrudate before it falls on the build platform.

In addition, during the collision and molding processes that will be considered later, the strand mainly experiences compression. Accordingly, the viscous resistance to stretching or compression is considered, while the viscous resistance to shear deformations is still neglected.

The expression of *F* was also derived in [27] for a Newtonian fluid. Following its derivation in [27] while considering that *µ* has the expression of Equation (1), we obtain an expression of *F* for a power-law non-Newtonian fluid, Equation (5):

(5) $$F=\frac{1}{2}\pi D^{2}[-(1+\frac{1}{2^{n}})L{\left|\frac{dU}{ds}\right|}^{m-1}\frac{dU}{ds}+\frac{\gamma }{R}]$$

According to the relations in Equation (2) and the value of *H*, we obtain the following scaling relations, Equation (6):

(6) $$\frac{U_{e}{}^{m}}{H^{m}}\;\sim \;20^{m}>1,\frac{\gamma }{DL}\;\sim \;0.01,\;\frac{\rho U_{e}{}^{2}}{L}\;\sim \;10^{-5},\;\frac{\rho gH}{L}\;\sim \;10^{-3}$$

All numbers in Equation (6) have the same unit of 1/s*^m^*. In the first relation, as *m* > 0, *20^m^* > 1. The second, third, and fourth relations in Equation (6) represent the relative effects of the surface tension, inertia, and gravity with the viscous force, respectively. According to these relations, these three effects can be neglected in comparison with the viscous effect.

In addition, at a scaling level, we obtain, Equation (7):

(7) $$\frac{dU}{ds}\;\sim \;\frac{U_{e}}{H},\;{\left|\frac{dU}{ds}\right|}^{m-1}\frac{dU}{ds}\;\sim \;\frac{U_{e}{}^{m}}{H^{m}},\;\frac{d\psi }{ds}\;\sim \;\frac{1}{H},\;\sin\psi \;\sim \;1,\;\cos\psi \;\sim \;1$$

Using Equations (5) and (7), both Equations (3) and (4) can be scaled to Equation (8):

(8) $$\frac{\rho U_{e}{}^{2}}{L}\sim -2(1+\frac{1}{2^{m}})\frac{U_{e}{}^{m}}{H^{m}}+\frac{\gamma }{DL}+\frac{\rho gH}{L}$$

Considering Equation (6), the second term in Equation (8) is considerably larger than the other three terms, which indicates that these three terms can be neglected in this equation. Consequently, Equations (3) and (4) are simplified to Equations (9) and (10)

(9) $$\frac{dF}{ds}=0$$

(10) $$F\frac{d\psi }{ds}=0$$

The solution to the set of Equations (5), (9), and (10) can be obtained by, Equations (11)–(14):

If

(11) $$\frac{d\psi }{ds}\neq 0$$

(12) $$U\left(s\right)=U_{e}$$

in case

(13) $$\frac{d\psi }{ds}=0$$

we obtain

(14) $$\psi \left(s\right)=\frac{\pi }{2},\;\frac{dU}{ds}=c$$

where *c* is a constant.

*ABC* and *JBCI* are, respectively, the side and cross-sectional views of the compressed portion of the extrudate at its contact region with the substrate (Figure 2c). Terms *F_x_* and *F_y_* denote the reaction forces applied by the substrate to the extrudate along the *x* and *y* directions, respectively, $l_{0}$ is the distance between *A* and *B*, while $F_{a}$ and $F_{b}$ are the axial forces at the two ends of the extrudate. The two ends may have different temperatures and thus different consistency indices. Therefore, when Equation (5) is used to determine $F_{a}$ and $F_{b}$, the corresponding values of *L* may be different. As discussed in Section 2, the time limit ensures that the temperature difference between the two ends of the extrudate is small, such that the consistency indices at these ends have the same order of magnitude. The corresponding consistency indices are not distinguished thereafter.

As what was done in [39], considering the balance of linear momentum on the portion of the extrudate from the nozzle to the end of the contact region (Figure 2c), Equations (15) and (16):

(15) $$F_{x}=\frac{\rho \pi D^{2}U_{s}{}^{2}}{4}+F_{b}$$

(16) $$F_{y}=F_{a}+\frac{\rho \pi D^{2}U_{e}{}^{2}}{4}-G$$

where *G* denotes the weight of this portion of the extrudate. 

Further, we analyze the balance of energy during the collision. In this process, the bottom of the extrudate’s cross-section is compressed. Therefore, the work is mainly carried out by *F_y_* along the vertical direction. It is, Equation (17):

(17) $$W\;\sim \;F_{y}\delta_{1}$$

The viscous dissipation is, Equation (18) [9]

(18) $$E=\int_{0}^{t_{c}}\int_{V}ødVdt\approx øVt_{c}$$

where ø denotes the dissipation function, V is the characteristic volume of the extrudate’s compressed portion, and $t_{c}$ is the characteristic time of formation of the compressed portion. At the contact region, the extrudate is impacted mainly by the stage along the *y* direction. Therefore, we focus only on the viscous dissipation along this direction. Consequently, we obtain, Equations (19)–(21):

(19) $$ø\;\sim \;L{\left|\frac{dU}{dy}\right|}^{m+1}$$

*V* can be expressed as

(20) $$V\;\sim \;l_{0}l_{1}\delta_{1}.\;$$

*t_c_* is estimated by the travel time of the extrudate through the contact region,

(21) $$t_{c\;}\sim \;\frac{l_{0}}{U_{s}}.$$

## 4. Results and Discussions

We consider that the deposition includes two processes: collision and molding. During the collision, only the bottom portion of the extrudate has contact with the substrate. It may be compressed. However, the top portion is not, since it does not have direct contact with the substrate during the collision. After the collision, the extrudate goes through the molding process. The substrate and nozzle serve as bottom and top parts of the mold, respectively. In the molding process, both top and bottom portions of the extrudate may be compressed.

We analyze two cases according to the relation between $U_{e}$ and $U_{s}$ (Table 1). In Cases I and II, $U_{e}\leq U_{s}$ and $U_{e}>U_{s}$, respectively. We investigate two subcases in either case. In the first subcase, after the collision, the thickness of the extrudate is less than *H*. However, it is larger than *H* in the second subcase. Therefore, in the first subcase, the extrudate only collides with the sustrate, while it is not molded. In the second subcase, it is further molded after the collision.

If the extrudate is not compressed by either substrate or nozzle during its deposition, its cross-sections are still circular in shape. According to the law of conservation of mass, the corresponding diameter, $D_{e}$, meets the following relation, Equation (22):

(22) $$D_{e}\;=\;D{\left(\frac{U_{e}}{U_{s}}\right)}^{\frac{1}{2}}$$

### 4.1. Case I.1

In this subcase, we assume that, Equation (23)

(23) $$D_{e}-\delta_{1}\leq H<2D$$

As indicated in [27], before the extrudate collides with the substrate, it has a catenary shape (Figure 3a). This is validated by our experimental result (Figure 3b). Thus, Relation (11) can be used. Therefore, the extrudate has a constant speed of $U_{e}$ before the collision, which implies that $\frac{dU}{ds}=0$. Using Equation (5), we obtain, Equations (24)–(26):

(24) $$F_{a}=2\gamma \pi D$$

Subsequently, using Equations (6) and (24), according to Equation (16)

(25) $$F_{y}=2\gamma \pi D+\frac{\rho \pi D^{2}U_{e}{}^{2}}{4}-G$$

Furthermore, using Equations (6) and (25), according to Equation (17),

(26) W ~ 0

Along the *y* direction, the maximum compressed size of the extrudate is $\delta_{1}$. The corresponding characteristic speed is $\frac{\delta_{1}}{t_{c}}$. Accordingly, $\frac{dU}{dy}\;\sim \;\frac{\frac{\delta_{1}}{t_{c}}}{\delta_{1}}$ = $\frac{1}{t_{c}},$ which, by Equation (21), yields, Equations (27)

(27) $$\frac{dU}{dy}\sim \;\frac{U_{s}}{l_{0}}$$

Using Equation (27), according to Equation (19), Equation (28)

(28) $$ø\;\sim \;\frac{LU_{s}{}^{m\;+\;1}}{l_{0}{}^{m\;+\;1}}$$

In addition, as what was done in [7], according to the geometric relation in the region of *JBIC* (Figure 2c), Equation (29)

(29) $$\delta_{1}\approx \frac{l_{1}{}^{2}}{4D}$$

Using Equations (19)–(21) and (28), according to Equation (18), Equation (30)

(30) $$E\;\sim \;\frac{Ll_{1}{}^{3}}{D}\frac{U_{s}{}^{m}}{l_{0}{}^{m\;-\;1}}$$

Considering Equations (26) and (30), the balance of *W* and *E* leads to, Equation (31)

(31) $$l_{1\;}\sim \;0$$

The combination of Equations (29) and (31) yields,

(32) $$\delta_{1}\;\sim \;0$$

During the collision, as the top portion of the extrudate is not directly exposed to external forces, Equations (33) and (34):

(33) $$l_{2}\;\sim \;0$$

(34) $$\delta_{2}\;\sim \;0$$

According to Equations (31) and (33), Equation (34):

(35) $$l_{b}\;\sim \;0$$

Equations (31)–(34) indicate that, during the collision, the extrudate’s cross-section experiences negligible deformation. As such, after the collision, the extrudate still has circular cross-sections, whose diameters are $D_{e}$ (Figure 3b). This explains a result published in [15,22]. For fast printing with a large nozzle height, it was found numerically in [15] and experimentally in [22] that the PLA extrudate’s cross-section was almost cylindrical. In their case, $\frac{U_{s}}{U_{e}}=1$ and D<H. Accordingly, it belongs to Case I.1. Consequently, according to our results, the deposited strand should have almost circular cross-sections. The derived results for this subcase are also validated by experimental results. As shown in Figure 3c1–c5, when *H* was fixed to be 0.5 mm and $U_{e}$/$U_{s}$ ranged from 0.6 to 1.0, the deposited extrudates have approximately circular cross-sections.

### 4.2. Case I.2

In this subcase, we assume that, Equation (36)

(36) $$D_{e}>H$$

During the collision, although there is negligible compression, the speed at the centerline increases from $U_{e}$ to $U_{s}$, and the diameter of the extrudate is changed from *D* to $D_{e}$. If Equation (36) is satisfied, after the collision, the extrudate has a thickness larger than the nozzle height and it should go through a molding process (Figure 4a,b). The extrudate is approximately axisymmetric before the molding. Owing to this symmetry, the top and bottom portions should be compressed by the same degree during the molding, which implies that. Equations (37) and (38):

(37) $$\delta_{1}=\frac{D_{e}-H}{2}$$

(38) $$\delta_{2}=\frac{D_{e}-H}{2}$$

As in [15,22], the compressed cross-section is assumed to have an oblong shape, which is a flat cuboid with rounded edges (Figure 4b). Subsequently, by geometric analysis and with the assistance of Equation (22), Equations (39) and (40)

(39) $$l_{1}=\frac{\pi }{4}\left(\frac{U_{e}}{U_{s}}\frac{D^{2}}{H}-H\right)$$

(40) $$l_{2}=\frac{\pi }{4}(\frac{U_{e}}{U_{s}}\frac{D^{2}}{H}-H)$$

Consequently, $l_{1}=l_{2}$, and Equation (41)

(41) $$l_{b}=\frac{\pi }{4}(\frac{U_{e}}{U_{s}}\frac{D^{2}}{H}-H)$$

The relations in Equations (39) and (40) are validated by experiments. The values of $l_{1}$ that are predicted using Equation (39) have a good agreement with the corresponding experimental results (Figure 4c). As further observed from Figure 4d1–f4, $l_{2}$ approximately equals $l_{1}$. The maximum difference is 16%, which appears in the sample shown in Figure 4f4.

### 4.3. Case II.1

In this subcase, Relation (23) is also assumed to hold true. However, different from that in Case I.1, the extrudate drops along the vertical direction, and then has a sharp turn as it lands on the contact region (Figure 5a). This has been validated by the experimental result (Figure 5b).

Since Relation (23) is satisfied, the top portion of the extrudate is not directly exposed to external forces during the collision. As in Case I.1, the top portion should still have an approximately circular profile. However, the bottom portion may be compressed (Figure 5c). Next, the focus is on finding its compressed width and depth.

In the vertical portion of the extrudate, Equation (13) is satisfied and U decreases from $U_{e}$ to $U_{s}$. Consequently, according to Equation (5), Equation (42):

(42) $$F_{a}\;\sim \;LD^{2}\frac{{\left(U_{e}\;-\;U_{s}\right)}^{m}}{H^{m}}$$

In the curved part of the extrudate, Equation (14) is satisfied. Therefore, in this part, the speed at the centerline remains $U_{s}$, which indicates that $\frac{dU}{ds}=0.$ Using Equation (5), we obtain, Equation (43):

(43) $$F_{b}\;\sim \;0$$

Subsequently, using Equations (6) and (42), according to Equations (15) and (16), Equations (44) and (45):

(44) $$F_{x}\;\sim \;0$$

(45) $$F_{y}\;\sim \;LD^{2}\frac{{\left(U_{e}\;-\;U_{s}\right)}^{m}}{H^{m}}$$

Equations (42)–(45) indicate that $F_{a}$ and $F_{y}$ are the two main forces applied on the suspended portion of the extruded strand. To obtain the balance of moments on this portion, the lateral area of the collision and cross-sectional area of the extrudate, which are the areas where $F_{a}$ and $F_{y}$ apply, should be approximately equal *D*. Therefore, we obtain Equation (46):

(46) $$l_{0}\;\sim \;D$$

The combination of Equations (17) and (45) yields, Equation (47)

(47) $$W\;\sim \;L\delta_{1}D^{2}\frac{{\left(U_{e}\;-\;U_{s}\right)}^{m}}{H^{m}}$$

In addition, as in Case I.1, $\frac{dU}{dy}$ can also be expressed by Equation (27). According to Equations (19), (46), and (27), ø can be expressed by Equation (28), while E can be expressed by Equation (30). 

Using Equations (29) and (46), according to Equations (47) and (30), the balance of *W* and *E* leads to Equation (48)

(48) $$l_{1}\sim \;\frac{D^{m\;+\;1}}{H^{m}}{\left(\frac{U_{e}}{U_{s}}-1\right)}^{m}$$

As shown in Figure 5d,e1–e5, this equation matches well with experimental tests. In calculating the relation between $l_{1}$ and $\frac{U_{e}}{U_{s}}$ using Equation (48), *m* was set to be 0.32 [2], D was 0.4 mm, and *H* was 0.5 mm.

According to Equation (29), Equation (49)

(49) $$\delta_{1}\;\sim \;\frac{D^{2m\;+\;1}}{H^{2m}}{\left(\frac{U_{e}}{U_{s}}-1\right)}^{2m}$$

Since the extrusion speed is higher than the print speed, the substrate applies a force during the collision to slow down the extrudate, making its speed match with the print speed. This force is represented by $F_{y}$. As observed from Equation (45), it is not negligible, and compresses the bottom of the extrudate.

As in Case I.1, since the top portion of the extrudate is not directly exposed to external forces during the collision, Equations (33)–(35) are also valid in Case II.1.

In addition, to validate Equation (45), $F_{y}$ was also determined, using a model that was originally applied for bi-directional rectilinear compression molding of polymers between two parallel plates [40]. In the compression model, the polymer melt was assumed to be a Newtonian fluid. According to this model, Equation (50)

(50) $$F_{y}\;\sim \;LV_{\mathrm{comp}}\frac{U}{h^{2}}$$

where $V_{\mathrm{comp}}$ is the volume being compressed, U is the clamping speed, and *h* is the gap between the two plates. In our case, $V_{\mathrm{comp}}$ is the volume of the compressed portion of the extrudate (Figure 2c). Thus, we have, Equation (51)

(51) $$V_{\mathrm{comp}}\sim l_{0}l_{1}\delta_{1}$$

Meanwhile, Equation (52)

(52) $$U\sim \;\frac{\delta_{1}}{t_{c}},\;h\sim \;\delta_{1}$$

Using Equations (51), (52), (21) and (49), according to Equation (50), Equation (53)

(53) $$F_{y}\;\sim \;LD^{2}\frac{U_{e}\;-\;U_{s}}{H}$$

It is identical to Equation (45), when *m* is set to be 1 in Equation (45) for a Newtonian fluid. As such, the compression model also validates our derived result.

### 4.4. Case II.2

In this subcase (Figure 6), we assume that Equation (54)

(54) $$D-\delta_{01}>H$$

In Equation (54), $\delta_{01}$ denotes the depth that the extrudate’s bottom is compressed during the collision. It equals $\delta_{1}$ of Case II.1, and also has the expression of Equation (49).

If Equation (54) is valid, the collided extrudate still has a thickness larger than the nozzle height. Consequently, a molding process follows the collision. The bottom portion of the extrudate is compressed during the collision, while the top portion is not. Accordingly, when the impacted extrudate is subjected to molding, the top and bottom portions of the extrudate are not geometrically symmetric. Consequently, they may be compressed by different degrees, leading to different flat areas at the top and bottom of the extrudate. Given that they are compressed by the same degree during the molding, we may still have $\delta_{1}\neq \delta_{2}$. For example, if the collided extrudate has a thickness just slightly larger than the nozzle height, both of its top and bottom portions are not compressed much during the molding process. As such, after the molding, $\delta_{1}\approx \delta_{01},$ $\delta_{2}\sim 0,$ and $\delta_{1}>\delta_{2}$. According to the above discussions, in general, $\delta_{1}\neq \delta_{2}$. Consequently, $l_{1}\neq l_{2}$, which is validated by our experimental results. As shown in Figure 6c1–d4, the difference between $l_{1}$ and $l_{2}$ ranges from 20 to 40%, except for the sample shown in Figure 6c3, which has a 4% difference between these two values.

A simple geometric analysis shows that the thickness of the molded extrudate should be equal to the nozzle height, which implies that, Equation (55)

(55) $$\delta_{1}+\delta_{2}=D_{e}-H$$

Accordingly, either $\delta_{1}$ or $\delta_{2}$ is smaller than $\frac{D_{e}-H}{2}.$ Considering Equation (29), $l_{1}$ or $l_{2}$ is smaller than $\sqrt{2D_{e}\left(D_{e}-H\right)}$. With the assistance of Equation (22), this result indicates that, Equation (56)

(56) $$l_{b}<{\left\{2D{\left(\frac{U_{e}}{U_{s}}\right)}^{\frac{1}{2}}[D{\left(\frac{U_{e}}{U_{s}}\right)}^{\frac{1}{2}}-H]\right\}}^{\frac{1}{2}}$$

## 5. Summary and Conclusions

In this study, we considered the compressed depths, flat widths, and bonding widths of the deposited extrudates in four different subcases. The derived relations are listed in Table 1. They have been validated, using our experimental tests (Figure 3, Figure 4, Figure 5 and Figure 6), experimental results of [2,22], numerical result of [15], and theoretical model of [40]. The obtained results are summarized as follows.

1. The deposition deformations of the extrudates do not depend on the extensional consistency index of the viscosity. This result agrees with the numerical result of [15] in the case of a Newtonian fluid. This result implies that, if the consistency index is beyond a certain value, which in our case has an order of 1000 Pa s*^m^* or higher, its variation has a negligible influence on the deformation. According to our model, this is valid if the travel time between the nozzle and substrate is small, such that the extrudate is approximately isothermal before its deposition. Consequently, the consistency indices at the two ends of the extrudate are in the same order. Subsequently, they are cancelled out in the consideration of the balance of energy during the deposition. If these consistency indices are considerably different, the deposition deformations should depend on their values. For example, in the limiting case, owing to the large travel time, if the extrudate has already solidified when it reaches the substrate, instead of deposition, it may simply bounce off the substrate.
2. In this work, the considered consistency index is in the order of 10,000 Pa s*^m^*. The relations listed in Table 1 can also be derived if it is in the order of 1000 Pa s*^m^*. However, when it is in the order of 100 Pa s*^m^*, as observed from the second relation of Equation (6), the effect of surface tension should be considered as well, since it is comparable to the viscous effect. The resulting relations may be different from the ones listed in Table 1.
3. As discussed in [15], the deposition deformations of the extrudates depend on the ratio between *U_e_* and *U_s_*, instead of only *U_e_* or *U_s_*. This point is validated by our experimental result on ABS, as well as that of [2] on ABS. When *U_e_* = *U_s_*, for three different values of *U_s_*, the corresponding values of $l_{b}$ were visually the same (see Figure 7 of [2]).
4. For *U_e_* ≤ *U_s_*, if $D_{e}$ < *H*, the extrudate is subjected mainly to the horizontal force applied by the substrate. The filament also increases its speed from *U_e_* to *U_s_* during the collision. The vertical reactions of the substrate (*F_y_*) are small, and the extrudate exhibits negligible compression. The extrudate does not go through the molding process. After deposition, it has an approximately circular cross-section.
5. For *U_e_* ≤ *U_s_*, if $D_{e}$ > *H*, the top and bottom of the extrudate are compressed between the nozzle and substrate by the same degree. Subsequently, as indicated in [15,22], which considered PLA, the cross-section of a deposited strand has an oblong shape. This special case is the optimal one in FFF, owing to its capability to have simple control of the bonding widths. Given that the nozzle height is fixed, the largest bonding width is obtained when *U_e_* = *U_s_*. According to Equations (41) and (22), it is $\frac{\pi }{4}(\frac{D^{2}}{H}-H)$.
6. When *U_e_* > *U_s_*, if $D_{e}$ < *H*, the extrudate is subjected mainly to the vertical force applied by the substrate. The extrudate speed initially decreases, and then is constant (*U_s_*). At the contact region with the substrate, the bottom of the deposited extrudate is compressed, while the top portion is not. The derived expression for *F_y_* agrees with the compression model [40], when it is simplified for a Newtonian fluid.
7. For *U_e_* > *U_s_*, if the collided extrudate has a thickness larger than the nozzle height, the top and bottom of the extrudate may be compressed between the nozzle and substrate by different degrees. Consequently, the top and bottom portions of the deposited strand may have different flat areas.

The derived analytic relations are simple (Table 1). For particular combinations of the processing parameters, they can be used to quickly predict the geometries of the deposited strands. If the extrusion speed is less than or equal to the print speed, and if the diameter of the extrudate is also larger than the height of the nozzle, controllable bonding widths can be produced between stacked strands. The largest bonding width is obtained when the extrusion speed is the same as the print speed. In the near future, we desire to find the following: (i) the numerical factor in the expression of $\delta_{1}$ in Case II.1; (ii) the expressions of $l_{1}$ and $l_{2}$ in Case II.2; and (iii) the deposition deformation of a single strand on a previously printed layer.

## Figures and Tables

**Table 1:** Summary of the flat widths, compressed depths, and bonding widths of the deposited strands in the four subcases.

Case	Conditions	*l*_1_	*δ*_1_	*l*_2_	*δ*_2_	*l_b_*	Cross-Sectional Profile after Deposition
I.1	$U_{e}\leq U_{s};$ $D{\left(\frac{U_{e}}{U_{s}}\right)}^{\frac{1}{2}}\leq H<2D$	0	0	0	0	0	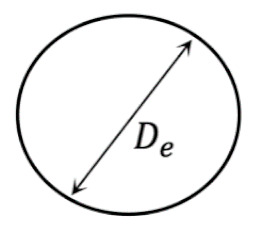
I.2	$U_{e}\leq U_{s};$ $D{\left(\frac{U_{e}}{U_{s}}\right)}^{\frac{1}{2}}>H$	$\frac{\pi }{4}(\frac{U_{e}}{U_{s}}\frac{D^{2}}{H}-H)$	$\begin{array}{c} {}[D{\left(\frac{U_{e}}{U_{s}}\right)}^{\frac{1}{2}}\\ -H]/2 \end{array}$	$\frac{\pi }{4}(\frac{U_{e}}{U_{s}}\frac{D^{2}}{H}-H)$	$\begin{array}{c} {}[D{\left(\frac{U_{e}}{U_{s}}\right)}^{\frac{1}{2}}\\ -H]/2 \end{array}$	$\frac{\pi }{4}(\frac{U_{e}}{U_{s}}\frac{D^{2}}{H}-H)$	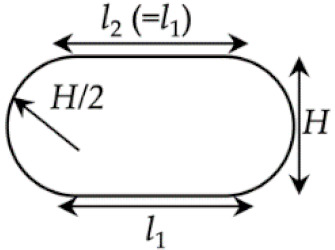
II.1	$U_{e}>U_{s};$ $D{\left(\frac{U_{e}}{U_{s}}\right)}^{\frac{1}{2}}-\delta_{1}\leq H<2D$	$\frac{D^{m+1}}{H^{m}}{\left(\frac{U_{e}}{U_{s}}-1\right)}^{m}$	$\delta_{1}\sim$ $\frac{D^{2m+1}}{H^{2m}}{\left(\frac{U_{e}}{U_{s}}-1\right)}^{2m}$	0	0	0	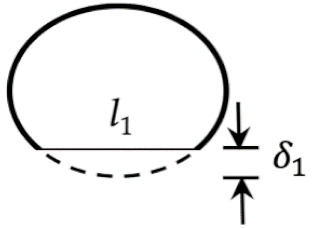
II.2	$U_{e}>U_{s};$ $D{\left(\frac{U_{e}}{U_{s}}\right)}^{\frac{1}{2}}-\delta_{01}>H$	N/I	$\delta_{1}+\delta_{2}=D{\left(\frac{U_{e}}{U_{s}}\right)}^{\frac{1}{2}}-H$	N/I	$\delta_{1}+\delta_{2}=D{\left(\frac{U_{e}}{U_{s}}\right)}^{\frac{1}{2}}-H$	$l_{b}<$ $\{2D{\left(\frac{U_{e}}{U_{s}}\right)}^{\frac{1}{2}}$ $.[D{\left(\frac{U_{e}}{U_{s}}\right)}^{\frac{1}{2}}-H]{\}}^{\frac{1}{2}}$	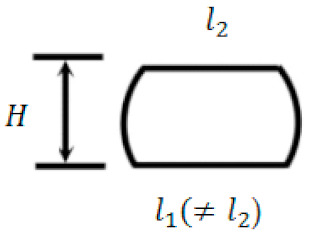

N/I: No Information.

## Nomenclature

Roman letters

$A_{d}$	Measured cross-sectional area of the deposited filament (${\mathrm{mm}}^{2}$)
*D*	Diameter of the just extruded strand (also diameter of nozzle) (mm)
*D*_e_	Diameter of the strand if there is no deposition deformation (mm)
E	Viscous dissipation
$F_{a}$	Axial force at the beginning of the suspended extrudate
$F_{b}$	Axial force at the end of the suspended extrudate
*F*	Axial force acting over the cross-section of the suspended extrudate
$F_{x}$	Reaction force applied by the substrate to the extrudate along the *x* direction
$F_{y}$	Reaction force applied by the substrate to the extrudate along the *y* direction
*g*	Acceleration of gravity (m/$s^{2}$)
*G*	Weight of the suspended extrudate
*h*	Gap between two molding plates (mm)
*H*	Distance between the nozzle head and the build platform (mm)
*K*	Shear consistency index (Pa s*^n^*)
*L*	Extensional consistency index (Pa s*^m^*)
$l_{0}$	Distance between *A* and *B*
$l_{1}$	Compressed width of the deposited filament’s bottom surface (mm)
$l_{2}$	Compressed width of the deposited filament’s top surface (mm)
$l_{b}$	Bonding width between two stacked filaments (mm)
*m*	Extensional thinning (or thickening) index
*n*	Shear thinning (or thickening) index
*Q*	Amount of material extruded per unit time
*s*	Arc length (mm)
$t_{c}$	Characteristic time of forming the compressed portion
$T_{r}$	Trouton ratio
*U*	Clamping speed in compression molding (mm/s)
$U_{e}$	Extrusion speed (mm/s)
$U_{s}$	Moving speed of the nozzle head (mm/s)
$U\left(s\right)$	Speed of the extrudate (mm/s)
V	Characteristic volume of the extrudate’s compressed portion
$V_{\mathrm{comp}}$	Compressed volume of the extrudate (${\mathrm{mm}}^{3})$
*W*	Work done by $F_{y}$
Greek letters	
$\psi \left(x\right)$	Inclination of the centerline to the horizontal surface
γ	Surface tension (mN m^−1^)
ρ	Mass density (kg m^−3^)
$\delta_{1}$	Compressed depth of the deposited filament’s bottom surface (mm)
$\delta_{2}$	Compressed depth of the deposited filament’s top surface (mm)
ø	Dissipation function
$\delta_{01}$	Compressed depth of the extrudate’s bottom during the collision (mm)
